# In Silico Ascription of Gene Expression Differences to Tumor and Stromal Cells in a Model to Study Impact on Breast Cancer Outcome

**DOI:** 10.1371/journal.pone.0014002

**Published:** 2010-11-19

**Authors:** Simen Myhre, Hayat Mohammed, Trine Tramm, Jan Alsner, Greg Finak, Morag Park, Jens Overgaard, Anne-Lise Børresen-Dale, Arnoldo Frigessi, Therese Sørlie

**Affiliations:** 1 Department of Genetics, Institute for Cancer Research, Division of Surgery and Cancer, Oslo University Hospital Radiumhospitalet, Oslo, Norway; 2 Institute of Clinical Medicine, Faculty of Medicine, University of Oslo, Oslo, Norway; 3 Department of Biostatistics, Institute of Basic Medical Sciences, University of Oslo, Oslo, Norway; 4 Department of Oncology, Aarhus University Hospital, Aarhus, Denmark; 5 Rosalind and Morris Goodman Cancer Centre, McGill University, Montreal, Canada; Technische Universität München, Germany

## Abstract

Breast tumors consist of several different tissue components. Despite the heterogeneity, most gene expression analyses have traditionally been performed without prior microdissection of the tissue sample. Thus, the gene expression profiles obtained reflect the mRNA contribution from the various tissue components. We utilized histopathological estimations of area fractions of tumor and stromal tissue components in 198 fresh-frozen breast tumor tissue samples for a cell type-associated gene expression analysis associated with distant metastasis. Sets of differentially expressed gene-probes were identified in tumors from patients who developed distant metastasis compared with those who did not, by weighing the contribution from each tumor with the relative content of stromal and tumor epithelial cells in their individual tumor specimen. The analyses were performed under various assumptions of mRNA transcription level from tumor epithelial cells compared with stromal cells. A set of 30 differentially expressed gene-probes was ascribed solely to carcinoma cells. Furthermore, two sets of 38 and five differentially expressed gene-probes were mostly associated to tumor epithelial and stromal cells, respectively. Finally, a set of 26 differentially expressed gene-probes was identified independently of cell type focus. The differentially expressed genes were validated in independent gene expression data from a set of laser capture microdissected invasive ductal carcinomas. We present a method for identifying and ascribing differentially expressed genes to tumor epithelial and/or stromal cells, by utilizing pathologic information and weighted t-statistics. Although a transcriptional contribution from the stromal cell fraction is detectable in microarray experiments performed on bulk tumor, the gene expression differences between the distant metastasis and no distant metastasis group were mostly ascribed to the tumor epithelial cells of the primary breast tumors. However, the gene PIP5K2A was found significantly elevated in stroma cells in distant metastasis group, compared to stroma in no distant metastasis group. These findings were confirmed in gene expression data from the representative compartments from microdissected breast tissue. The method described was also found to be robust to different histopathological procedures.

## Introduction

Female breast cancer counts for over 465 000 deaths annually world wide (approximately 1.300.000 new cases), and is the most common cancer type among women [1]. Earlier diagnosis and more efficient treatment strategies have reduced the mortality and lowered the risk for recurrence. Established molecular markers such as the estrogen receptor (ER), progesterone receptor (PgR) and human epidermal growth factor receptor 2 (*HER2/neu*) are considered routinely for treatment decisions [2].

Whole-genome DNA microarrays for gene expression have made it possible to unravel biological mechanisms underlying the disease at the transcriptomic level [3]. Microarray experiments allow for measurements and comparisons of gene expression within a sample as well as between multiple samples. For example, gene expression profiling has shown that breast tumors can be classified into subgroups displaying distinct characteristics with respect to both clinical markers and patient outcome [4]–[7], which emphasizes that breast cancer is a heterogeneous disease that should be treated accordingly. Specific gene signatures have been identified that correlate with different aspects of the disease, including two risk-predictors for distant recurrence that are currently being tested in large clinical trials [8]–[12].

Breast carcinomas are heterogeneous “organs” with a microenvironment of various cell types both within the tumor area and in the surroundings. Lymphocytic infiltrations, fibrosis, angio- and lymphanogenesis, and stromal cells (including fibroblasts, myofibroblasts and adipocytes), leukocytes, and myoepithelial cells may influence the development and further progression of the carcinoma cells [13]. Although there are studies showing that alterations and involvement of the microenvironment (stromal tissue) of breast carcinomas influence outcome [14], [15], most gene expression analyses have been performed using the bulk tumor, without further reference to the stromal content. Hence, without performing any cell selection (by microdissection) prior to gene expression analysis, the output data have contained transcriptomic information from all tissue components in the sample. The variable contribution from different cell fractions makes it difficult to state whether differences in gene expression between samples arise from distinct expression within the same cell type, and/or reflect the diversity in gene expression between various types of cells [16].

Cleator and colleagues [17] showed, using gene expression microarray analysis, that the stromal component of breast carcinomas influenced the prediction of therapy response. They emphasized the significant effect the non-tumor-cell content of breast cancer samples has on gene expression profiles. Furthermore, in a study of prostate cancer, Stuart and colleagues [18] linked, *in silico*, the relative content of tumor cells, benign hyperplastic epithelium, stroma, and dilated cystic glands in 88 prostate specimens to gene expression levels determined by microarray analysis. They were able to identify gene expression differences between non-malignant and malignant epithelial cells without being confounded by the gene expression of stromal cells. Since the content of tumor cells varies between patient samples, a threshold for minimum content of tumor cells within a sample is often applied. Such a threshold is quite arbitrary and would exclude potentially important cell type interactions.

In this study we utilized the histopathological information of tumor epithelial cell and stromal cell area-fractions to perform cell type-associated gene expression analyses of primary breast tumors. We compared a group of breast cancer patients who experienced distant metastasis (DM) with a group who experienced no distant metastasis (NoDM) during a median follow up period of >15 years. In this approach, using tumor epithelial cell-based weights, a sample where these cells cover a small percentage of the area of the tissue sample, will contribute less than a sample where tumor cells are more abundant when investigating gene expression differences originating in tumor tissue. We provide here an *in silico* method to ascribe differentially expressed gene-probes (DEGs) in primary breast tumors between patients experiencing DM and patients experiencing NoDM to either tumor epithelial and/or stroma cells.

## Results and Discussion

Initially a standard t-test was performed, referred to as the “un-weighted analysis”, between the DM and NoDM tumor groups in a set of primary breast tumor samples from 198 patients. Clinical characteristics are given in Table 1. The un-weighted test identified 182 differentially expressed gene-probes (DEGs) at a 5% FDR level (Table S1”). This gene-probe list was in the following referred to as the *UWA-list* (Un-weighted analysis-list). Similar results were obtained when using variations of the t-statistics, as presented by Tusher and colleagues [19].

**Table 1:** Clinical information.

			DM		NoDM	
p- value	Parameter		No.	%	No.	%
	All		118	60	80	40
0.692	Protocol b	CMF	55	47	35	44
	Protocol c	TAM	63	53	45	56
0.105	Radiotherapy	YES	54	46	46	58
		NO	64	54	34	42
0,054	Tumor Sixe	<20 mm	32	27	30	38
		20–50 mm	64	54	44	55
		>50 mm	22	19	6	7
0.000	Pos lymph nodes	None	8	7	3	4
		1–3	43	36	62	77
		>3	67	57	15	19
0.002	Histopathology	Ductal	90	77	63	86
		Nonductal	28	23	17	14
0.004	Malignancy	Grade 1	17	19	17	27
	(Ductal-only)	Grade 2	42	47	39	62
		Grade 3	31	34	7	11
0.008	ER-status	Neg	39	33	13	16
		Pos	79	67	67	84
		Unknown	0	0	0	0

The null hypothesis is that the probabilities for each outcome (DM or noDM) are independent of the state or treatment (as written in the column).

One sided Chi-square or Fisher exact test for 2 by 2 or 2 by three contingency tables.

### Testing the relationship between cell area fraction and overall expression

**Figure 1:**
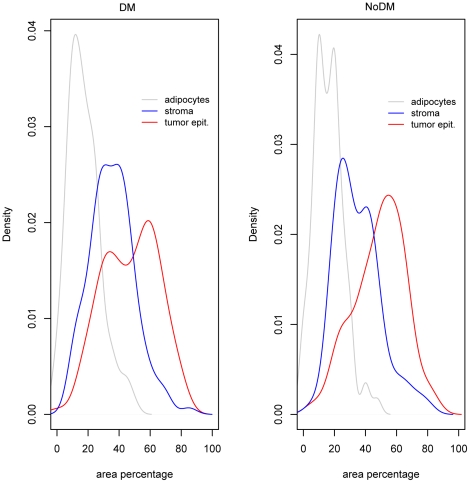
Cell type distribution between outcome groups. The density of the data was estimated by kernel smoothing where each point on the curve gives the probability (on the y-axis) of observing a given value x (of the x-axis). Percentages of the area of H&E sections of three different tissue components; tumor epithelial cells, stromal cells and adipocytes, grouped by clinical group. (DM = distant metastasis; NoDM = no distant metastasis).

For every tumor sample, histopathologic information regarding the content of different tissue components (tumor epithelium, stroma and adipocytes) was available (see Materials and methods).The distribution of the different tissue components was not significantly different (p-value 0.78 for tumor epithelial cells and 0.87 for stromal cells, Kolmogorov-Smirnov two-sample test) between the DM and noDM groups (Figure 1).

In order to test if the pathological numbers of the area fraction of tumor epithelial and stromal cells made an impact on the expression data we collected a list of genes reported to be stromal or tumor cell related in breast cancer by literature search [17], [20]. Two methods were applied to see how the expression of these genes was related to the pathological numbers in our data. In the first approach, we compared gene expression data from the 15 tumor pieces having the highest stroma cell area-fraction with the expression data from the 15 tumor pieces having the highest tumor epithelial cell area-fraction. By doing a SAM [19] between the high tumor epithelial cell - and high stroma cell area fraction samples, we obtained one list of genes whose expression were elevated in the samples with high tumor epithelial cell area-fraction (list A), and one list of genes whose expression were elevated in the samples with high stroma cell area-fraction (list B). In the second approach we calculated the Pearson correlation between the gene expression for all genes and the mean centered pathological numbers for stromal (list C) and tumor epithelial cell area-fractions (list D) (across all 198 samples). The majority of the genes extracted from the literature were confirmed in our data, thus indicating that the pathological scoring of tissue components was robust (see Table S2). This indicates that both tumor stroma and tumor epithelial cells contribute a measurable amount of mRNA transcripts into the gross gene expression signal from the bulk tumor. We were not able to identify any candidate markers for adipocytic expression.

### Robustness with respect to histopathology

The histopathological numbers utilized in the weighted analyses were crude estimates of area-fractions of tumor epithelial, stroma and adipocytic cells. In order to assess the robustness of the weighting approach in relation to the histopathological procedure, stereological point counting was performed additionally on a representative selection of the tissue sections. The numbers of area fraction from the stereological point counting were then utilized to calculate new weights for the sub-selection of samples. The new weights were compared to the initial weights used in the analyses. If the weights from the two methods were significantly different, then the output from the analyses would depend on the histopathological method applied. The first step of the validation was to test if the 109 samples selected for stereological point counting were representative for the whole set of 198 samples. None of the three statistical tests which compared the mean area-fraction for tumor epithelial, stroma and adipocytes respectively in the two sets of samples were found to be significant. Therefore we concluded that the 109 samples were representative for the whole data set. The second step was to compare the area-fraction numbers of tumor-epithelial, stroma and adipocytes in the 109 samples common for the two histopathological procedures. The differences in area fractions between the two procedures were highly significant for all three area fractions estimated. The stereological point counting tended to give a lower scoring of tumor epithelium and adipocytes and a higher scoring of stroma area-fraction, compared to the initial histopathological estimates. Hence, these tests revealed significant differences in the exact area fraction values measured with the two methods which are in accordance with previous publications determining that semiquantitative measurements tend to produce an overestimation as compared to stereology [21]. However, in the weighted analyses it is not the proportion of tumor epithelial versus stroma cell area fraction within one sample that is important. But rather how the numbers of tumor epithelial and stroma area-fraction in the individual samples are in respect to the total number of tumor epithelial and respectively stroma area-fraction in all samples (in this case the group of individuals who did or did not experience DM). Therefore, new tumor and stroma weights were computed based on the number of area fraction from the stereological point counting. Then the weights were re-scaled by setting the level of transcription of adipocytes to null as described in the “statistical methods” section. The new weights were compared to the original ones in a paired t-test. The obtained p-values for the paired t-test were close to one for both the tumor and stroma weight comparisons, indicating no differences between the weights derived from the two histopathological approaches. Hence, it appears from these analyses that the weighting method is robust to variations in histopathological procedures.

### Weighing under different assumptions of adipocyte transcription

**Figure 2:**
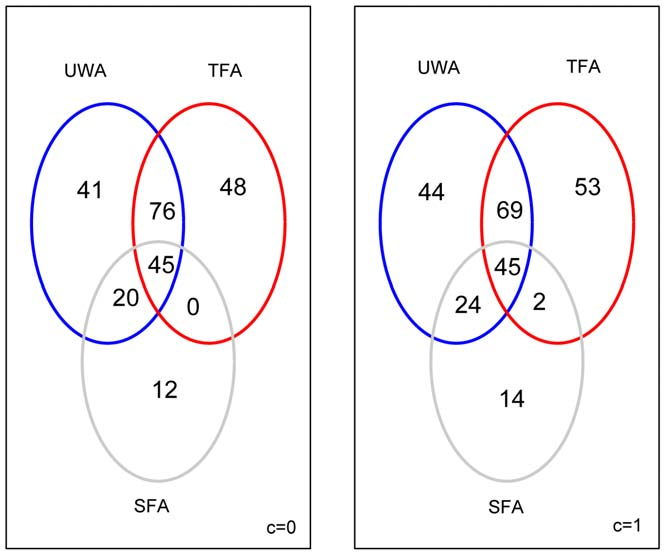
Overlap of differentially expressed gene-probes for different assumptions of adipocyte transcription. The Venn diagrams illustrate the sets of differentially expressed gene-probes with their intersections. The number of probes are given in each set, identified in unweighted (blue), tumor-focused (red) and stroma-focused (grey) analyses, respectively, when adipocytes contribute no transcript (c = 0), and when adipocytes contribute equally to tumor and stroma cells (c = 1).

The level of transcriptional contribution from adipocytes, compared to tumor epithelial and stromal cells in a heterogenous compartment is uncertain. Therefore, the statistical analyses were first carried out under various assumptions on the relative transcriptional efficiency of adipocytes compared to the two other tissue components. In the first series of analyses, DEGs between the DM and NoDM groups were identified under two different assumptions of mRNA levels in adipocytes (denoted by *c*): Firstly, we assumed that total adipocyte transcription level was equal to stroma cells and tumor epithelial cells (*c = 1*); secondly, we assumed no transcription in adipocytes (*c = 0*). The weighted analyses were divided into *tumor-focused analyses* (TFA) and *stroma-focused analyses* (SFA). In TFA, each sample was weighed proportionally to the area fraction tumor epithelium of that tumor sample, while in SFA, by the stromal cell percentage of that tumor sample. Thus, higher impact in the statistical analysis was given to samples containing more tumor epithelium or stromal cells, respectively. The TFA and the SFA respectively returned 169 and 77 DEGs, under the assumption of no transcription in adipocytes (c = 0). When the adipocyte transcription was weighed equally as for tumor epithelium and stroma (c = 1), the numbers of DEGs found between DM and NoDM tumor groups were 169 in the TFA and 85 in SFA, respectively (Figure 2). All the DEGs were obtained after multiple testing adjustments (5% FDR).

There were several overlapping gene-probes in the lists of DEGs. Fourty-five gene-probes were found in common between UWA and the lists from both weighted analyses (TFA and SFA) when *c = 0*. An identical number of overlapping probes between UWA and the weighted analyses were also identified under the assumption of *c = 1* (Figure 2). The intersection of these two core sets of 45 gene-probes was 31. Hence, these 31 gene-probes were identified by the UWA as well as the SFA and TFA under both assumptions on adipocyte transcription (*c = 0* and *c = 1*) and were among the most differentially expressed gene-probes between the two outcome groups. In addition, most of the 182 probes in UWA (representing 181 different genes) were also detected in the weighted analyses (141 for *c = 0* and 138 for *c = 1*). The remaining probes (41 and 44 when *c = 0* and *c = 1*, respectively) were not identified in the weighted analysis at 5% FDR but appeared as significant at 10 or 20% FDR. It is interesting that both the TFA and SFA identified probes that appeared as statistically non-significant in the standard un-weighted analysis. These were 48 and 55 tumor-associated probes (when *c = 0*, and *c = 1*, respectively) and 12 and 16 stroma-associated probes (for *c = 0* and *c = *1, respectively) (Figure 2).

**Figure 3:**
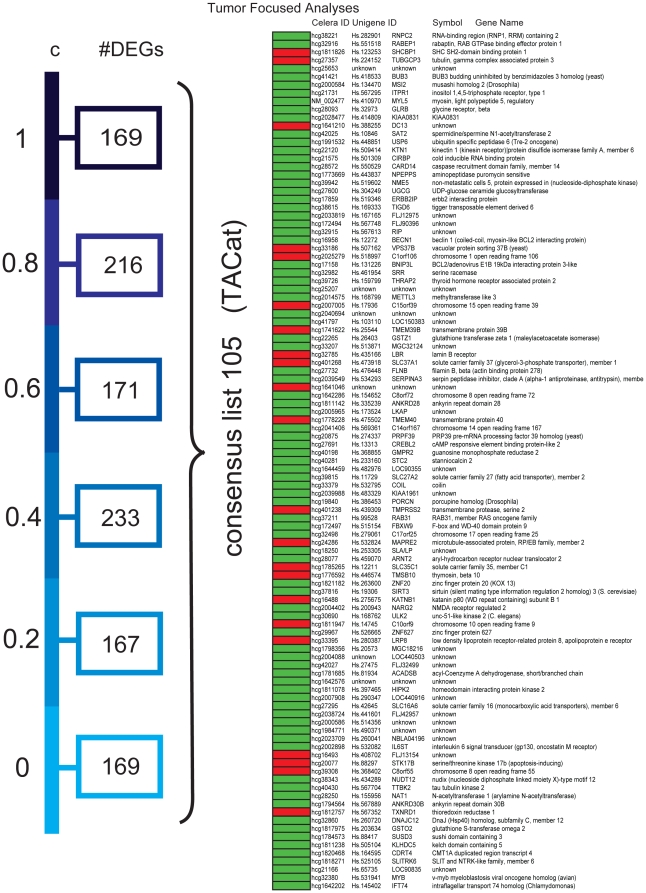
Differentially expressed gene-probes identified in the tumor-focused analysis for various assumptions of adipocyte transcription. A set of DEGs was identified from each of six analyses for different assumptions of c. A consensus list of 105 probes was categorized. Red boxes: up in DM ; Green boxes: up in NoDM. (TACat - tumor associated consensus-list with adipocyte transcription).

**Figure 4:**
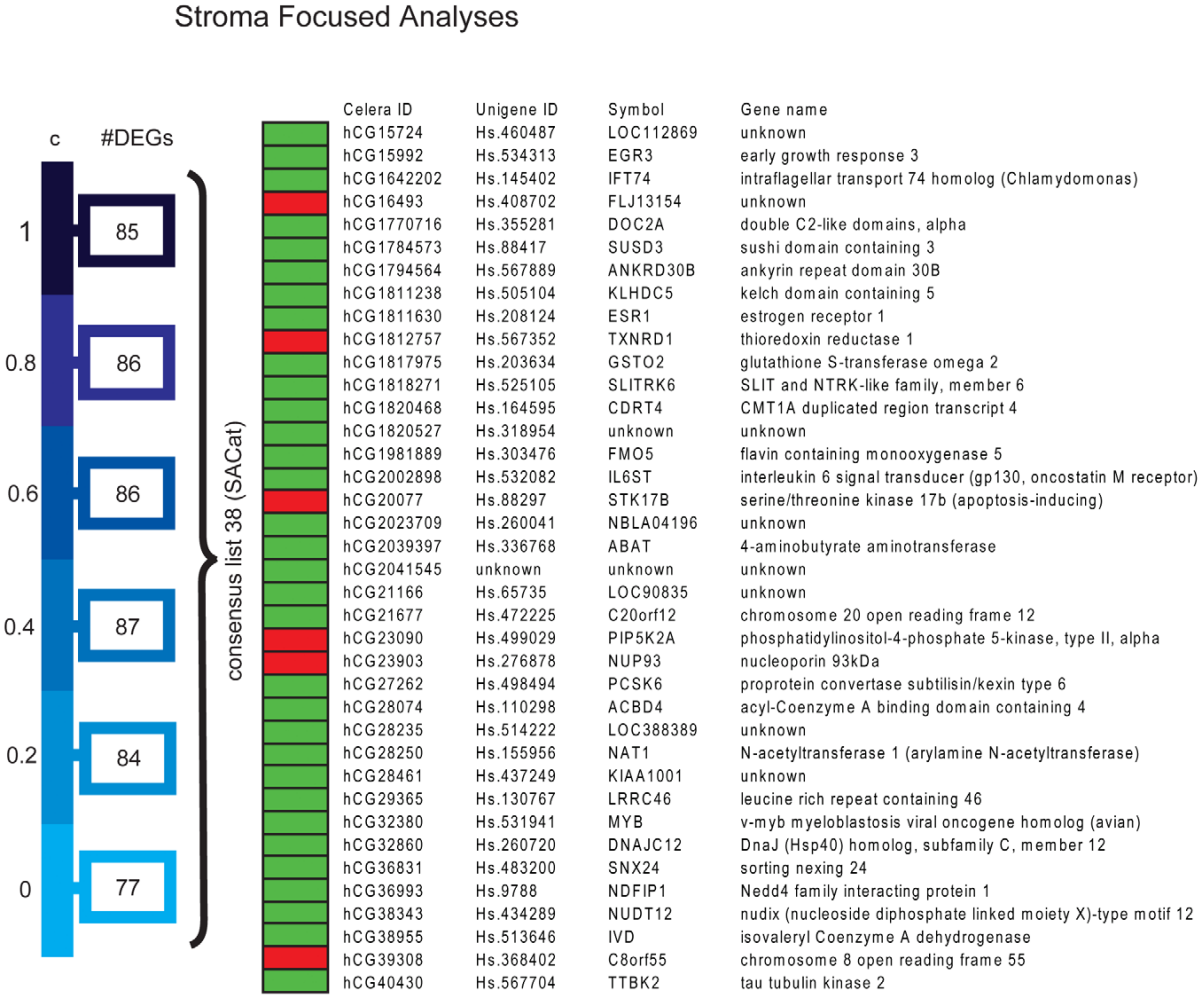
Differentially expressed gene-probes identified in the stroma-focused analysis for various assumptions of adipocyte transcription. In the stroma-focused analysis, a **set** of genes for each assumption of c was identified and a consensus list of 38 probes was determined. Red boxes: up in DM ; Green boxes: up in NoDM. (SACat - stroma associated consensus with adipocyte transcription).

Lists of DEGs identified through weighing by tumor epithelium and stroma content were then generated separately for other various assumptions of adipocyte transcription levels from *c = 0 to 1 (c = 0, 0.2, 0.4, 0.6, 0.8 and 1)*. In summary, in the TFA there were 105 DEGs which appeared for all the selected adipocyte transcription levels (tumor associated consensus-list with adipocyte transcription, *TACat* ) (Figure 3), while there were 38 DEGs in the stroma consensus list (stroma associated consensus with adipocyte transcription, *SACat*) (Figure 4). Based on these analyses and the comparison of all generated lists of DEGs under various assumptions of adipocyte transcription level, we decided to proceed the analyses with c = 0, as the impact on the differentially expressed gene-probes between DM and noDM under the various assumptions on adipocyte transcription was minor.

### Weighing under different assumptions of stromal cell transcription

**Figure 5:**
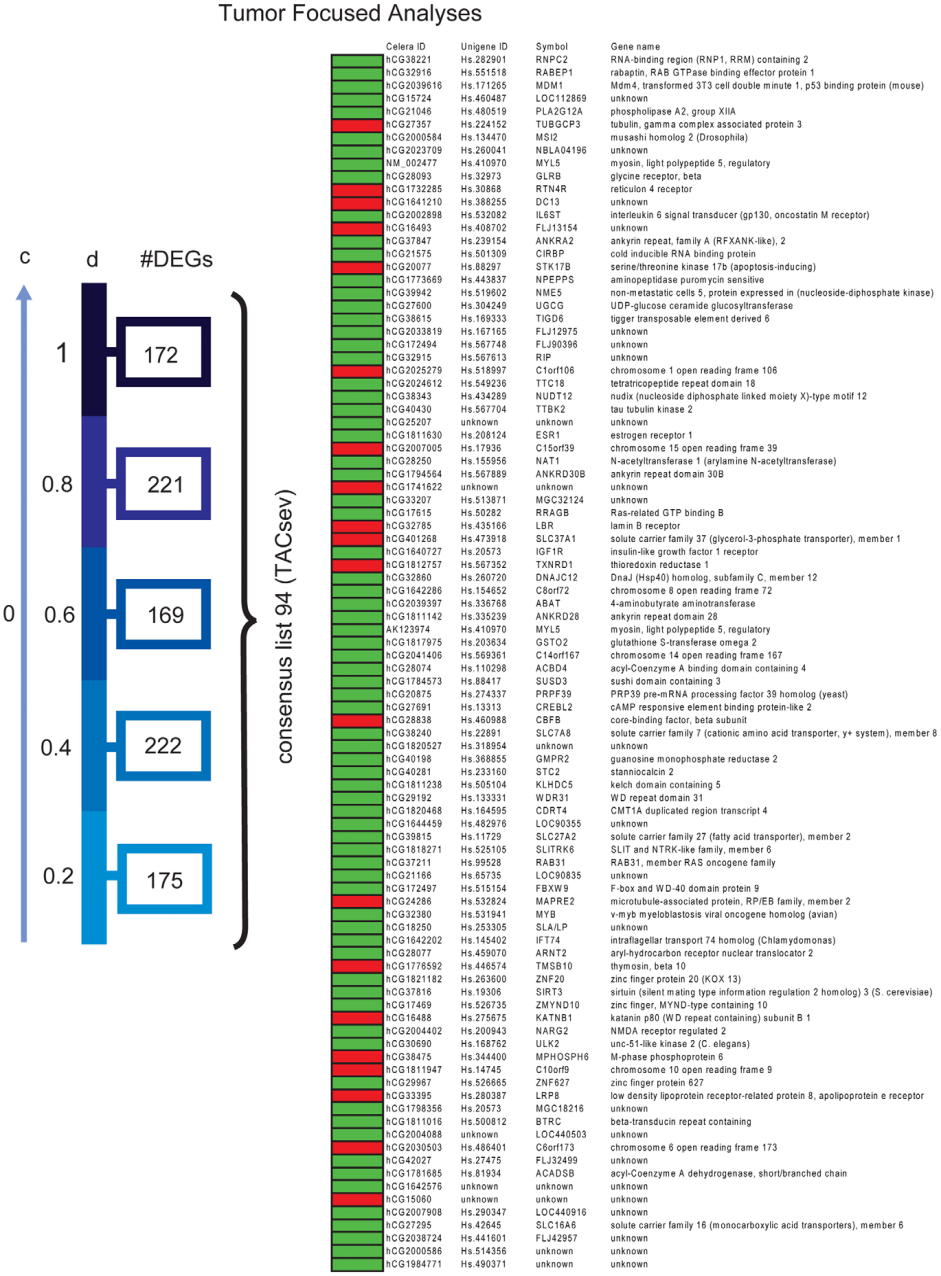
Differentially expressed genes identified in the tumor-focused analysis for various assumptions of stroma transcription. Similarly, a set of DEGs was identified for each of six tumor-focused analyses, under various assumptions on the contribution of transcripts from stroma. A consensus list of 94 probes was determined. C = 0 for these analyses and the area of tumor and stroma cells was re-scaled to sum to 100. Red boxes: up in DM ; Green boxes: up in NoDM. (TACsev - tumor associated consensus-list stroma expression varied).

Even though the contribution from fat cells on transcriptional differences between two outcome groups in our analyses seems to be minimal, this may not be the case for stromal cells. In fact, it has been suggested that genes expressed in tumor-related stroma may predict disease outcome for breast cancer patients [15]. In the next series of analyses of differentially expressed gene-probes between tumors from patients experiencing DM or NoDM during follow up, we performed both tumor-epithelium-focused and stroma-focused analyses under various assumptions on mRNA level from the stromal compartment. Similarly to the above analyses, we varied the stromal expression strength *d*, in five intervals from 0.2 to 1 (d = 0 was not selected as it would be equivalent to the UWA), and identified five lists of DEGs in the tumor-weighted analysis (Figure 5). If the true stromal transcription level (compared with tumor epithelium) was known, the list of DEGs identified using the true d would be selected. Since the true d is unknown, these five lists were interpreted together. When d = 1, stromal and tumor epithelial cells were assumed to have equal levels of mRNA transcription. In the tumor-focused analyses, the contribution of each sample then equaled the samples' tumor epithelium percentage. For each selected value of d, the individual sample weights were re-scaled (see Materials and methods). A low value of d assumes low mRNA transcription level in stromal cells compared to tumor cells, and the weight of each sample was therefore increased in a TFA and decreased in a SFA. For different values of d, the re-scaling of the weights gives stronger impact to samples with a high stroma percentage in a TFA as d is selected from 1 towards 0.2, since a low d assumes that the mRNA transcription mainly comes from tumor epithelial cells. Hence, the list of gene-probes from the TFA when d = 1 is less likely to contain DEGs originating from stromal tissue than when d = 0.2, because samples that consisted of mostly tumor epithelial cells counted more in the TFA when d = 1. Finally, in order to distinguish DEGs most likely to originate from tumor epithelium, we need to subtract the DEGs identified in the TFA with the DEGs identified in the SFA.

A set of 94 DEGs (tumor associated consensus-list stroma expression varied, *TACsev*) was identified regardless of the assumption on the level of stroma cell transcription d (Figure 5). The *TACsev-*list is less likely to contain DEGs originating from the stromal compartment since it includes the DEGs identified with d = 1.

**Figure 6:**
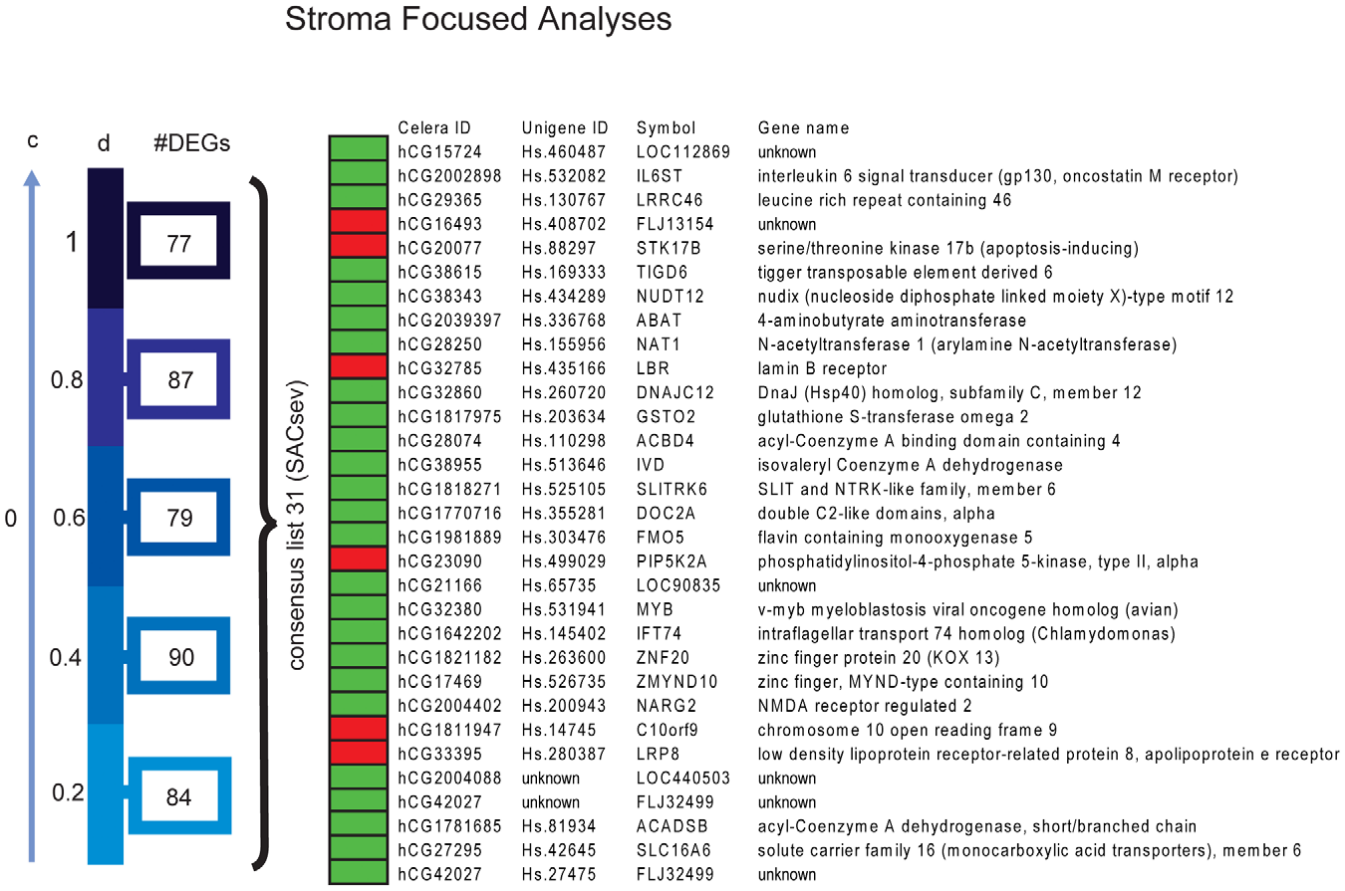
Differentially expressed gene-probes identified in the stroma-focused analysis for various assumptions of stroma transcription. For various assumptions on stromal cell contribution, a set of DEGs was identified. C = 0 for these analyses and the area of tumor and stroma cells was re-scaled to sum to 100. A consensus list of 31 probes was determined. Red boxes: up in DM ; Green boxes: up in NoDM. (SACsev - stroma associated consensus list stroma expression varied).

Further, in the stroma-weighted analysis, again under various assumptions on the level d of transcription from stromal cells, the number of DEGs varied as *d* was increased from 0.2 to 1. Here, a set of 31 DEGs (stroma associated consensus list stroma expression varied, *SACsev*) was always identified regardless of the assumption on the level of transcription of stromal cells (Figure 6). The SFA is less prone to identify DEGs originating from tumor epithelial cells, since samples were weighted according to stroma content. In these analyses, a sample counted more if it consisted of more stroma (and hence less tumor cells) while it counted less if there were few stromal cells and proportionally more tumor epithelium, for each d from 1 to 0.2.

**Figure 7:**
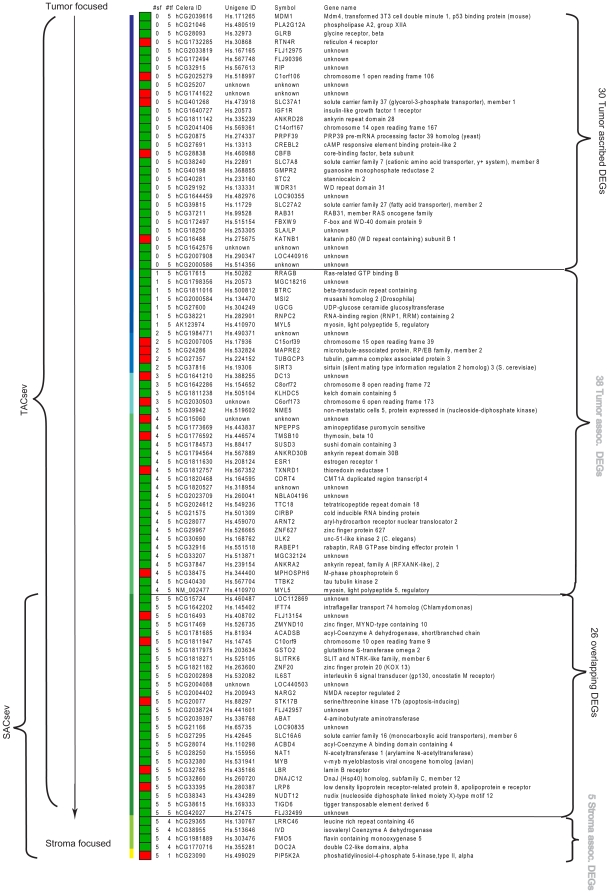
Differentially expressed genes in TACsev and SACsev identified by the weighted analyses. The most repeatedly identified probes in either every tumor focused analysis or every stroma focused analysis for different values of d (assumed stromal transcription level). The ‘#tf’ and ‘#sf’ columns list in how many tumor focused and stroma focused analyses, respectively, the corresponding DEGs were identified. The colored vertical bar goes from darker blue (highly tumor associated) to lighter blue as the DEGs are identified in more stroma focused analyses (for different values of d), to green for DEGS found in both SFA and TFA, and finally yellow for stroma associated DEGs identified in fewer tumor focused analyses. Red boxes: up in DM ; Green boxes: up in NoDM.

One of the values of d should be close to reflecting the true stromal contribution, but as this is unknown and *SACsev* represents the intersection of all the selected values of d, we expect most true DEGs to be in this list. These DEGs are likely to originate from stromal cells; if the same DEG also appears in *TAC*sev, it could be an indication that the tumor focused analyses wrongly ascribed the differential expression of this gene-probe to tumor cells. The DEGs in *TACsev* that are *not* in *SACsev* are hence more likely to be tumor epithelial cell-related. Because of these conceptual aspects, we focused on the identified DEGs that composed the intersections (consensus lists) from the analyses based on all 5 values of d *(TACsev* and *SACsev*), since these are most likely to withhold the true d (assuming the stroma transcription level was not less then <20% of tumor cell transcription level, as a conservative hypothesis) (Figure 7). We interpret these gene-probes as the most robustly identified DEGs between the DM and NoDM group, and the most biologically interesting. Finally, one important issue is that gene-probes whose expression profiles show extreme differences originating from tumor cells between the two outcome groups, might still have been identified in the stroma focused analyses even if tumor epithelium contributed less (and vice versa).

### Biological interpretation of the consensus gene lists

We identified 68 DEGs in *TACsev* (94 DEG-list) that were not identified in *SACsev* (31 DEG-list). However, 38 of the 68 DEGs were identified by one or more of the stroma weighted analyses under different assumptions of stromal transcription level and could therefore not be ascribed exclusively to tumor epithelial cells (Figure 7). These 38 DEGs are therefore referred to as “tumor cells associated”. We do propose that the differences in expression between DM and NoDM for the remaining 30 unique gene-probes in *TACsev* descended from tumor cells, since these were not identified by any of the five stroma weighted analyses. Among these were the insulin-like growth factor 1 receptor *(IGF1R)*, cAMP responsive element binding protein-like 2 *(CREBL2)*, and RAB31, member RAS oncogene family *(RAB31)*.

In the *SACsev* list we could not ascribe any DEGs purely to stroma cells since there were no DEGs between DM and NoDM that were not identified in at least one of the tumor-focused analyses (i.e. *TACsev*). In fact, four out of five SACsev gene probes not found in TACsev were still identified by four out of five tumor focused analyses when the contribution of stroma expression was varied (Figure 7). The fifth gene, phosphatidylinositol-4-phosphate 5-kinase, type II, alpha (*PIP5K2A*) was only identified by one of the five *TACsev-*analyses. These DEGs are therefore referred to as “stroma cells associated”. However, it is interesting to note that these genes were identified by the tumor focused analyses for the lower values of d, hence, differences in gene expression from these five genes, and *PIP5K2A* in particular, might mainly be associated to stromal cells. *PIP5K2A* is part of the v-akt murine thymoma viral oncogene homolog 1 (*AKT1*) signaling pathway [22] and was higher expressed in tumors from patients who experienced distant metastasis. *PIP5K2A* has been reported to be involved in differentiation and motility; hence it might influence the metastatic process. The remaining four DEGs of the *SACsev* list that were not in *TACsev* were flavin containing monooxygenase 5 (*FMO5*), isovaleryl Coenzyme A dehydrogenase *(IVD)*, leucine rich repeat containing 46 *(LRRC46)*, and double C2-like domains, alpha *(DOC2A)*, all found to be lower expressed in the DM group. Longer relapse-free survival has previously been reported for patients with higher expression of *FMO5* [23] which is in line with the observed expression of *FMO5* in our study. Isovaleryl-CoA dehydrogenase (*IVD*) is a mitochondrial matrix enzyme that catalyzes the third step in leucine catabolism, and was found to be lower expressed in rats injected with estradiol valerate [24], which might indicate that this gene is downregulated by estrogen. In our study, *IVD*-expression was lower in the DM-group, in which tumors also showed lower expression of estrogen receptor 1 (*ESR1*) when compared to the NoDM group. It is on the other hand, questionable how comparable these two studies are. Regarding *LRRC46*, a recent study reports that this gene was expressed higher in estrogen receptor positive breast cancer tumors which are comparable to what was observed in our study [25]. *DOC2A* was reported to be highly expressed in brain cells where the protein was found to interact with Ca2+ and phospolipid in neurotransmission [26], and to our knowledge, no apparent connection between this gene and metastasis in breast cancer has previously been reported. There were two different *DOC2A*-probes present on the microarray used in this study, and only one of these showed significant difference in expression between the DM and NoDM groups. These probes might represent different splice variants, and need to be further validated.

There were 26 DEGs in common between the two consensus lists of DEGs (*TACsev*, and *SACsev*) (Figure 7). We consider these probes most robust in being differentially expressed between DM and NoDM since they are identified in every instance of cell type focus under different assumptions of stroma transcription. Among these 26 probes we did find known breast cancer related genes such as Arylamine N-acetyltransferase-1, (*NAT1*) [27], and v-myb myeloblastosis viral oncogene homolog (*MYB*), both reported earlier as a potential prognostic markers in estrogen receptor positive breast tumors [28]. Both *NAT1* and *MYB* were found to be lower expressed in the DM group in our data. Another gene of interest (also lower expressed in DM) is the zinc finger, MYND-type containing 10 (*ZMYND10*) gene, which has been suggested to act as a tumor suppressor gene in nasopharyngeal carcinomas [29]. All 26 DEGs in common between *TACsev and SACsev* lists were also found by the UWA. This is as expected since the criteria of the 26 gene list is that the DEGs were identified by all values of d in both TFA and SFA, also including the TFA with d = 0,2, a test being close to the UWA. Thus, the expression differences between the DM and NoDM groups for these probes seem to be sufficient to overshadow the weighing by either cell type. Conceptually, the cell type ascription of these DEGs could be explained by different scenarios; a) ascribed to tumor epithelial cells assuming the expression of tumor epithelial cells overshadows any effect of stroma; b), ascribed to stromal cells by assuming the expression differences in stroma were adequately profound to be detectable in a stroma focused analysis at low values of d (stroma-weighted test with d = 0,2) or c) these genes are differentially expressed between DM and NoDM in both cell type compartments. Analysis of expression differences for these genes in the validation data set indicates that a) is the most likely scenario of the three. However, we did validate one gene (ABAT) that was significantly differentially expressed between DM and NoDM in both tumor epithelial and tumor stroma.

### Overlap between weighted and un-weighted tests

**Figure 8:**
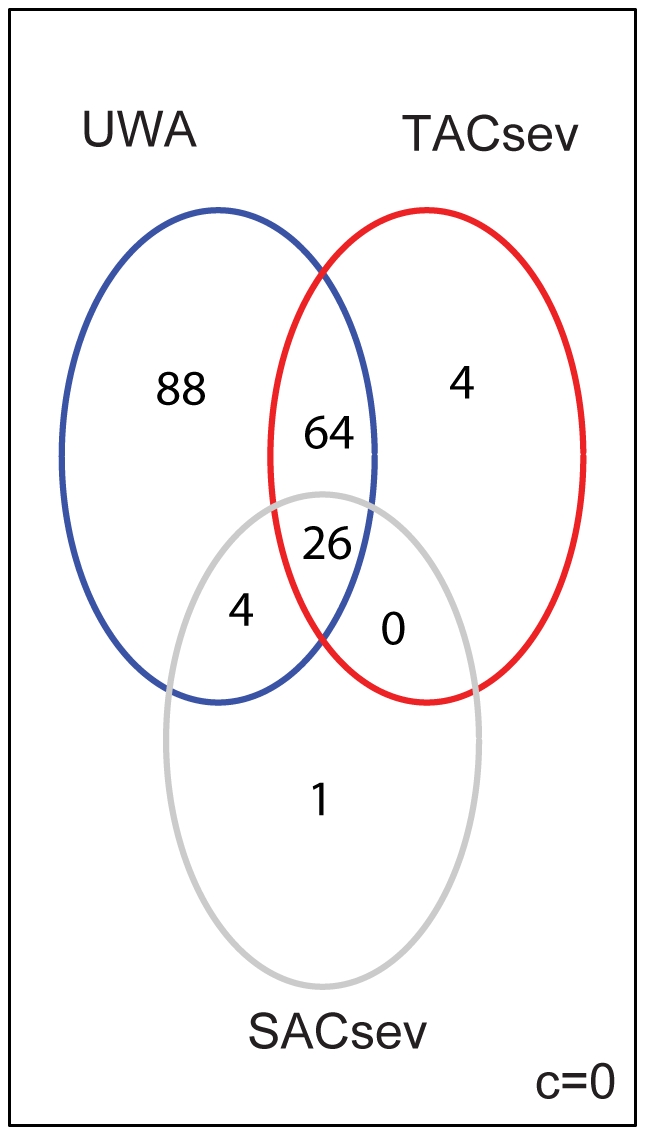
Overlap of differentially expressed gene-probes for the consensus lists (SACsev and TACsev) and UWA. The overlap of probes identified by the unweighted analysis and the intersection of all tumor weighted analyses and stroma weighted analyses with values of d from 1 to 0.2, under the assumption of no transcription in adipocytes. The majority of genes, 64 of the 68 tumor cell ascribed (30) and associated (38), and four of the five stromal cell associated are identified in the UWA.

A total of five probes were identified in the consensus lists from the weighted analyses that were not among the DEGs from the un-weighted statistics (Figure 8). One probe from *SACsev* list (*PIP5K2A*), and 4 probes from the *TACsev* list (*FLJ90396*, *PRPF39*, *Unassigned* with homology to *Rab22*, and *MAPRE2*). *MAPRE2* (microtubule-associated protein, RP/EB family member 2) was found highly expressed in the DM group, and has been suggested (due to homology) to be involved in tumorigenesis of colorectal cancers and proliferation control in normal cells [30]. There is no reported function for the remaining three genes. Furthermore, we identified 88 probes (representing 88 genes) in UWA which were neither in *TACsev* nor *SACsev*. However, when we considered all 312 DEGs identified in the weighted analyses for all values of *d* between 0,2 - 1, there were 137 genes that were not present in the 182 UWA-list, and conversely, only 6 probes (6 genes) in the UWA list that were not present in any list from the weighted analyses (see Table S3).

### Validation

In order to validate the tumor stromal and tumor epithelial ascribed (and associated) gene expression differences between the DM and NoDM groups, the expression of the genes in the consensus gene list (Figure 7) was tested in an independent data set. In the validation set, tumor epithelial cells and tumor stroma cells from 50 and 61 breast cancer patients, respectively, were isolated using laser capture microdissection (LCM) [15].

To assess the differences in expression of each gene between the DM and NoDM group a simple t-test was utilized. Out of the 30 tumor ascribed DEGs (in figure 7) 21 had available gene expression in the tumor-epithelial LCM validation set. Seven out of the 21 genes were found significantly differentially expressed (p<0.05) between DM and NoDM patients in the validation set with coherent up and downregulation in the respective clinical groups. Nine additional genes showed up and downregulation in the same direction as what was found in the initial analyses compared to the initial analyses. However, these genes did not reach significant p-values in the validation set (0.05<p<0.5). The remaining 5 genes showed no difference between DM and NoDM (see Table S4).

Out of the 38 tumor associated DEGs 29 had available gene expression in the tumor-epithelial LCM validation set. Eight out of the 29 genes were found significantly differentially expressed (p<0.05) between DM and NoDM patients in the validation set with coherent up and downregulation in the respective clinical groups compared to the initial analyses. Fourteen additional genes showed up and downregulation in the same direction as what was found in the initial analyses. However, these genes did not reach significant p-values in the validation set (0.05<p<0.5). The remaining 6 genes showed no difference between DM and NoDM (one gene had non-significant opposite regulation in the validation set).

Out of the 26 overlapping DEGs 19 had available gene expression in the tumor-epithelial LCM and the tumor-stroma validation sets. Eight out of the 19 genes were found significantly differentially expressed (p<0.05) between DM and NoDM patients in the tumor-epithelial validation set. The eight genes had coherent up and downregulation in the respective clinical groups compared to the initial analyses. Six additional genes showed up and downregulation in the same direction as what was found in the initial analyses. However, these genes did not reach significant p-values in the validation set (0.05<p<0.5). The remaining 5 genes showed no difference between DM and NoDM (one gene had non-significant opposite regulation in the validation set). In the tumor-stroma validation set, one gene (ABAT) was found significantly differentially expressed (p<0.05), and was among the eight significant genes in the tumor-epithelial set. Eight genes showed up and downregulation in the same direction as what was found in the initial analyses. However, these genes did not reach significant p-values in the validation set (0.05<p<0.5). The remaining 10 genes showd no difference between DM and NoDM (one gene had non-significant opposite regulation in the tumor-stroma validation set).

Out of the five stroma ascribed genes, one gene (PIP5K2A) was found significantly differentially expressed in the tumor-stroma validation set. This gene was not significantly differentially expressed in the tumor-epithelial validation set. Thus, the PIP5K2A seem to be validated as a gene being significant differentially expressed in the tumor-stroma cells but not in tumor-epithelial cells of DM vs NoDM patients. This differentially expressed gene was also the most stroma related as it was only identified for one value of d in the tumor focused analyses (Figure 7). One gene (LRRC46) was found significantly differentially expressed in the tumor-stroma validation set but in the opposite direction of the initial analysis. The expression values of this probe had low variance in the validation set, and might not be a functional probe (Table S4).

### Impact of weighing

The unweighted approach returned a list of DEGs in which there were varying numbers of false positives at any chosen FDR. When the statistics was weighed by cell type content under various assumptions of transcription levels in adipocytes and stromal cells, we identified a set of re-occurring genes that were differentially expressed between the DM and NoDM group. DEGs identified under different statistical scenarios appear more robust and are more likely true positives. However, the effect of weighing samples differently in the analyses is similar to decreasing the sample size of the dataset, since each sample count less than 1. Hence, some of the differences among the lists of DEGs could be due to a sample size effect and not the weighing it-self. The different assumptions on transcription levels in adipocytes showed a miniscule impact on the numbers of identified DEGs, and were thus set to zero, although we acknowledge that there are studies emphasizing the importance of adipocytes in breast cancer [31], [32].

It should be noted that we, on the basis of weighing, can not state whether or not a gene is expressed to a higher extent in tumor epithelial cells compared to stromal cells. We merely propose to which cell type the expression *differences* (between DM and NoDM) of a gene could be ascribed or associated.

### Conclusions

We present an *in silico* method for utilizing histopathological information on tissue content of tumor biopsies in the statistical calculations of differentially expression of genes in microarray data. This approach was used to identify genes significantly differentially expressed between tumors from patients who experienced distant metastasis and tumors from those who did not. The weighted analyses were utilized to ascribe and associate gene expression differences between the two patient groups to tumor epithelial and stromal cells. Although the stromal transcription contribution was measurable in data obtained from grossly dissected tumor tissue, the differences in gene expression between DM and NoDM seem mainly to be related to tumor epithelial cells, and the majority of these genes can also be identified by the unweighted bulk tumor expression. The analyses also aided in identifying a set of core genes which seems to be profoundly different in their expression patterns between the two groups, independent of the cellular constitution. Thus, genes reappearing in the weighted analyses under different assumptions of cellular contribution to the overall transcription level have a higher probability of being true findings.

## Materials and Methods

### Ethics statement

The study was approved by the national ethics committee (Ethical Commitee for Aarhus county) and by “Datatilsynet” (The Data Inspectorate - an independent administrative body under the The Ministry of Government Administration and Reform). Oral informed consent was mandatory, and a procedure approved by the ethical committee at that time (1982–1987).

### Patient material

The 198 breast tumor samples with high quality gene expression data utilized in this study were part of 267 fresh frozen tumor samples (stored at −80°C in Denmark) available after total mastectomy surgery. The tumor samples were originally stored for the purpose of estrogen receptor staining in the Danish Breast Cancer Group 82 b and c cohort (DBCG82b&c). DBCG82 b and c studies comprise a collection of tumor tissues from 3,083 high-risk Danish breast cancer patients diagnosed in the period 1982–1990. High-risk was defined as positive lymph nodes and/or tumor size larger than 5 cm and/or invasion of tumor to surrounding skin or pectoral fascia. Total mastectomy with partial axillary dissection was performed on all women, and a median of seven lymph nodes was removed from the axilla.

Premenopausal women (DBCG b protocol) were randomized to receive radiation therapy and CMF (cyclophosphamide, methotrexate, fluorouracil; eight cycles) or only CMF chemotherapy (9 cycles) [33]

Postmenopausal women (DBCG c protocol) were randomized to receive radiation therapy+tamoxifen (30 mg daily for 1 year) or tamoxifen alone

### Follow-up

Patients were followed routinely at regular intervals the first 10 years or until the first recurrence, death or new primary cancer. Recorded endpoints were locoregional recurrence (LRR), distant metastases (DM) and contralateral breast cancer (CBC) [34].

### Sample handling

Fresh frozen tumor samples (approximately 5–10 mm^3^) from a subset of 267 out of the 3,083 patients in the DBCG82 b and c protocols were available for this study. All tumor samples were cut in three and the centre section was used for total RNA extraction whereas the two flanking pieces were used for histopathological analyses using Haematoxylin & Eosin (HE) staining (Figure S1). Approximately 40 mg of tissue was used for total RNA extraction using the Qiagen Midi kit Extraction column procedure (Qiagen) after homogenisation using the Mixer Mill (MM301, Retsch). RNA quality was assessed using the NanoDrop instrument (concentration and purity), and the Agilent Bioanalyser instrument (degradation). Samples showing a degradation factor >20% were excluded from further analyses: the remaining 218 RNA samples of good quality were stored at −80°C.

### Histopathology

Haematoxylin & Eosin (HE)-stained sections were prepared from the two flanking tissue pieces facing the centre section used for RNA extraction (Figure S1), and HE sections were used for evaluating the cellular composition of the tissue sample. The tissue components evaluated were: 1) stroma, primarily comprising tumor-related stroma but also normal, surrounding stroma including vessel walls and lymphocytes. 2) epithelium, comprising tumor epithelium and possible normal epithelium entrapped in the tumor area or lying subjacent to the invasive component, and finally, 3) adipocytes entrapped in or surrounding the tumorarea. The area fractions from two sections per patient sample were estimated by a pathologist (JMN), and validated by stereological point counting [35]. A grid consisting of 20–70 points (depending on size of the tissue sample) were superimposed on each tissue section using a projection microscope and the entire section was evaluated by moving the grid in a random, systematic way. Hits on folds, not allowing proper morphological recognition of the structures, were excluded. Area fractions were calculated as number of points hitting profiles divided by total number of tissue points.

Most sections were entirely comprised of invasive tumor, and in these the amount of normal tissue stroma was negligible.

### Microarray analysis

The microarray system used in the study was the Applied Biosystem Human Genome Survey Microarray version 2.0. These are whole genome arrays spotted with 32878 probes covering 29098 genes. The platform utilizes chemiluminescence labeling in a single channel system. The probes are 60-mers, mostly mapped within 1500 bases of the 3′ end of the source transcript, and were designed to match common regions of multiple alternative transcripts for a particular gene. For some genes there are multiple probes, although 87% of the genes are targeted by a single probe. In addition to the human gene probes, the microarray contains numerous internal control probes for monitoring the experimental steps in the amplification, labeling and hybridization procedure. A second set of control probes is present for intra array normalization purposes (spot to spot normalization). These probes are 20-mers printed alongside the gene-probes in every spot. The shorter control-probes are complementary to a fluorescently labeled control sequence that is added to the hybridization solution. The fluorescent signal emitted should be identical for every spot; the gene-spot signal is normalized parallel to the difference in the fluorescent signal in the same spot compared to all the other spots [36]. 500 ng of total RNA was used as input in the amplification and labeling procedure and 10 µg of labeled and amplified cRNA was hybridized onto the array for 16 hours followed by washing and signal detection. Twenty arrays were removed due to too low present call or failed out on the amplification efficiency control probes, 7 samples with no available histopathology were excluded from the weighted analyses (but included in the unweighted) giving a total of 191 unique samples with good quality expression data and histopathologic information.

### Data processing and normalization

First, flagged intensities and probes with signal to noise ratio below 3 were removed from the raw data. Next, the number of probes was further reduced to 17910, by removing those that had missing values in more that 80% of the samples. The quantile normalization procedure was applied using ABarray library in R and the missing values were imputed using the k-nearest neighbors algorithm of the smida library in R. After re-scaling the expression on each array so that they sum to 1 a log2 transformation was applied. Data is accessible at NCBI GEO database accession GSE24117.

### Statistical analysis

Across the whole dataset, the percentages of stroma cell area varied from 5% to 85%, for tumor cells; from 5% to 85%, and for adipocytes; from 0% to 50%. A two-sample Kolmogorov-Smirnov test was used to compare the distribution of the different cell types in the DM and NoDM groups [37].

To incorporate the information on cell type abundance into the testing procedure, the tumor-weighted or stroma-weighted mean expression of the gene was computed instead of the arithmetic mean, for both groups of patients (DM and NoDM) as 
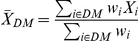
 and 
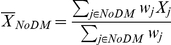
 where the sums go over all samples in each cohort and the weight *w_i_* and *w_j_* is the mean percentage (average of the two sections per sample) of area covered by tumor (or stroma) in the sample. The difference of the means was taken and standardized giving the statistics 
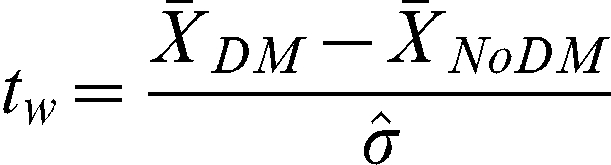
, where 
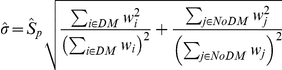
. We assumed that the variability in the gene expression in both cohorts is equal and estimated the pooled variance by 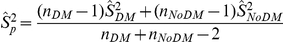, 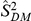, and 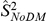, being the estimated variances of the respective cohorts and 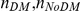 their sizes. The p-values were computed by performing permutation test and after a 5% FDR adjustment for multiple testing, different sets of differentially expressed genes (DEGs) between the two cohorts were identified. The un-weighted analysis was performed by setting the weights to 1 which is equivalent the standard t-test with equal variances.

The tumor-focused and the stroma-focused analyses were carried out under various assumptions. In the first series of analyses we assumed that the total gene transcription of the three tissue components (tumor, stroma and adipocytes) occurred at an equal level. However, due to uncertainty around the transcription level of adipose tissue, the weighted analysis was repeated for various relative efficiencies *(* ***c*** *)* of transcription of adipocytes compared to tumor cells. For different values of ***c*** ranging from 0 to 1, the adipocytes percentage area *(* ***a*** *)* was scaled to ***ac*** and the percentages of area covered by tumor and stroma were re-scaled so that they summed up to 100. The re-scaled tumor *(* ***t′*** *)* and stroma ***(s′*** *)* weights were computed as *t′ = t/(t+s+ac)* and *s′ = s/(t+s+ac)*, where ***t***, ***s*** and ***a*** are respectively, tumor, stromal and adipocyte cell area proportions in the original data and ***c*** the relative level of transcription of adipocytes compared to the other two tissue components.

The analyses carried out for the other selected values of *c* gave different sets of genes, differentially expressed between patients who developed distance metastasis and those who did not. In both series of analyses (tumor-weighted and stroma-weighted, for different levels of *c*), some genes seemed to be consistently differentially expressed regardless of the assumption on the level of transcription of adipocytes. Next, the level of transcription of the adipocytes was set to zero and the cell type-weighted analysis was performed under different assumptions on the level of transcription ***d*** of stroma cells compared to tumor cells. Analogous to the first series of analyses, for each level of *d* the percentages of area covered by tumor and stroma were re-scaled to sum up to 1. These new weights were computed ad *t″ = t′/(t′+s′d)* and *s″* = (*t′+s′*)*−t″*.

### Validation data

Gene expression data and associated clinical information on metastatic disease were available from microdissected tumor stroma and tumor epithelium from patients with invasive ductal carcinoma [15]. The tumor epithelial dataset included 61 patients with either distant metastatic disease (16) or no local or distant metastatic disease (45); the tumor-stroma dataset included 50 patients with either distant metastatic disease (10) or no local or distant metastatic disease (40). (Out of the 50 patients with available tumor-stroma gene expression two patients were not among the 61 with available tumor-epithelial expression data). The samples in the validation set had been analyzed on Agilent Whole Human Genome 44K arrays and hence, probes were matched between the Applied Biosystem whole genome survey microarrays and the Agilent platform by gene symbols. For genes being represented by more than one probe, the probe that matched the target RefSeqs was selected. In cases where multiple probes matched the RefSeq, the probe with highest variance across the sample cohort was selected. Out of the 99 DEGs in SACsev and TACsev combined, we were able to identify 74 matching genes in the validation set (24 out of 31 DEGs in SACsev and 69 out of 94 DEGs in TACsev) (Table S5 and S6).

## Supporting Information

Figure S1 RNA extraction. Fresh-frozen tumor piece cut in three, a, b and c. Sections cut from pieces a and c for pathological estimation of area percentages of adipocytes, stromal cells and carcinoma cells. Centre piece b was used for total RNA extraction and microarray analyses.
(0.27 MB PDF)

Table S1 Genes associated with tumor epithelial and stromal expression. List of genes (Gene symbol and Celera Gene ID) with reported cell type association in literature, were compared to the fold change (and local FDR) from a SAM analysis between the 15 samples with the highest tumor cell percentage and the 15 samples with the highest stroma cell percentage. The Pearson correlations (and p-value) for each gene to the tumor and stroma cell percentage across all 198 samples are also included.
(0.04 MB XLS)

Table S2 UWA geneprobe list. List of DEGs from the unweighted analysis, 182 gene-probes differentially expressed in tumor samples from patients who experienced distant metastasis compared with those who did not.
(0.04 MB XLS)

Table S3 DEGs under various assumptions of d. Lists of all DEGs found in the various weigthed analyses under different assumptions of the contribution of mRNA transcription from stroma.
(0.10 MB XLS)

Table S4 Validated DEGs in LCM stroma and tumor epithelial expression data. List of 74 genes that were matched to the stroma and tumor epithelial expression validation set. Left columns in the split table show the original regulation of genes in DM vs. NoDM found in the stroma and tumor weighted analyses, respectively. The next column shows the regulation seen in the validation sets (only for those with a p-value<0.5). The significant differences in the validation sets are highlighted in red.
(0.03 MB XLS)

Table S5 LCM Stroma. List of all gene probes with gene expression data for the matching 74 genes in the LCM stroma. Left column annotates no distant metastasis = 0 and metastasis = 1.
(0.15 MB XLS)

Table S6 LCM Epi. List of all gene probes with gene expression data for the matching 74 genes in the LCM tumor epithelial. Left column annotates no distant metastasis = 0 and metastasis = 1.
(0.18 MB XLS)
