# Supplementary material for: In Silico Ascription of Gene Expression Differences to Tumor and Stromal Cells in a Model to Study Impact on Breast Cancer Outcome
Source: PLoS One. 2010 Nov 19;5(11):e14002. doi: 10.1371/journal.pone.0014002 (PMC2988804; doi:10.1371/journal.pone.0014002)
Supplement: Figure S1 — RNA extraction. Fresh-frozen tumor piece cut in three, a, b and c. Sections cut from pieces a and c for pathological estimation of area percentages of adipocytes, stromal cells and carcinoma cells. Centre piece b was used for total RNA extraction and microarray analyses. (0.27 MB PDF) [file pone.0014002.s001.pdf]

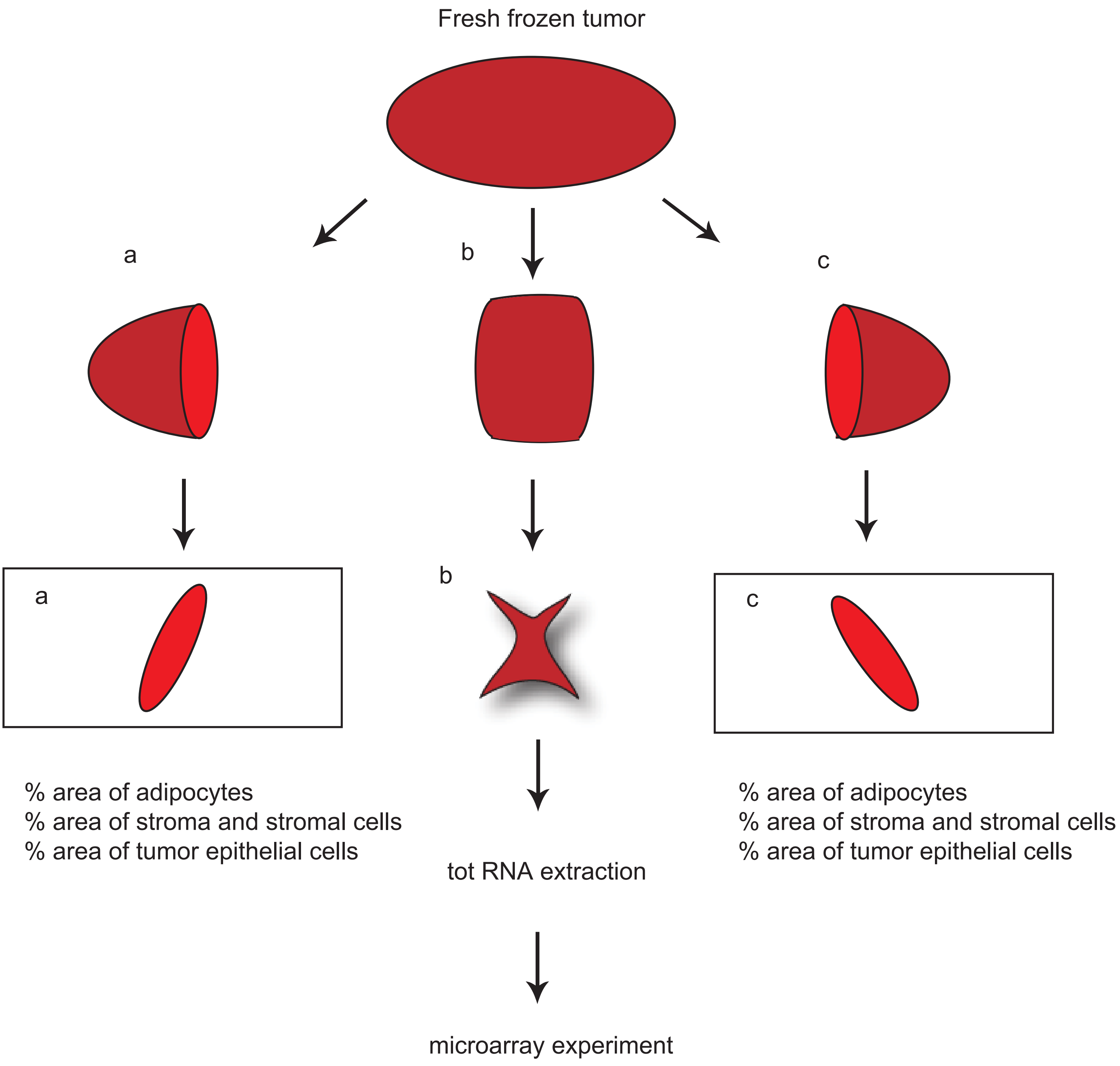
Fresh frozen tumor
a
b
c
a
b
c
% area of adipocytes
% area of stroma and stromal cells
% area of tumor epithelial cells
tot RNA extraction
% area of adipocytes
% area of stroma and stromal cells
% area of tumor epithelial cells
microarray experiment
